# Macrophage-related molecular subtypes in lung adenocarcinoma identify novel tumor microenvironment with prognostic and therapeutic implications

**DOI:** 10.3389/fgene.2022.1012164

**Published:** 2022-10-03

**Authors:** Heng Wen, Hanjian Chen, Liwei Xie, Zetao Li, Qian Zhang, Qiping Tian

**Affiliations:** ^1^ Department of Anesthesiology, The First Affiliated Hospital, Zhejiang University School of Medicine, Hangzhou, China; ^2^ Department of Anesthesiology, Jincheng People’s Hospital, Jincheng, China

**Keywords:** macrophages, lung adenocarcinoma, tumor microenvironment, prognostic model, molecular subtypes

## Abstract

**Background:** Lung adenocarcinoma (LUAD) is a life-threatening malignant tumor, contributing for the largest cancer burden worldwide. Tumor microenvironment (TME) is composed of various immune cells, stromal cells and tumor cells, which is highly associated with the cancer prognosis and the response to immunotherapy, in which macrophages in TME have been revealing a potential target for cancer treatment. In this study, we sought to further explore the role of macrophages in LUAD progression and establish a risk model related to macrophages for LUAD.

**Methods:** We explored immune-related pathways that might be affected by counting positively associated genes in macrophages. Molecular typing was also constructed by mining macrophage-associated genes with prognostic value through COX regression and other analyses. RiskScore prognostic models were constructed using lasso regression and stepwise multifactorial regression analysis. The differences on clinical characteristics among three subtypes (C1, C2, and C3) and RiskScore subtypes were analyzed in TCGA dataset. Immunological algorithms such as TIMER, ssGSEA, MCP-Counter, ESTIMATE, and TIDE were used to calculate the level of difference in immune infiltration between the different subtypes. The TCGA mutation dataset processed by mutect2 was used to demonstrate the frequency of mutations between different molecular subtypes. Finally, nomograms, calibration curves, and decision curves were created to assess the predictive accuracy and reliability of the model.

**Results:** The C1 subtype demonstrated the best prognostic outcome, accompanied by higher levels of immune infiltration and lower mutation frequency, while the majority of patients in the C1 subtype were women under 65 years of age. Myeloid-derived suppressor cell (MDSC) scores were higher in the C3 subtype, suggesting a more severe immune escape, which may have contributed to the tumor evading the immune system resulting in a poorer prognosis for patients. In addition, our RiskScore prognostic model had good predictive accuracy and reliability.

**Conclusion:** This paper provides a study of macrophage-related pathways, immunosuppression, and their mechanisms of action in lung cancer, along with targets for future treatment to guide the optimal treatment of lung cancer.

## Introduction

The incidence of lung cancer is currently on the rise worldwide, and as demonstrated by the latest epidemiological surveys, lung cancer is the most prevalent malignancy among men and its incidence is second only to breast cancer among women. However, it is still the most prevalent cause of cancer-related fatalities (Sung et al., 2021) as it accounts for more than 25% of all cancer-related deaths and is one of the most aggressive tumors (Siegel et al., 2020). Lung cancer is divided into two groups, distinguished by histological characteristics: small cell lung cancer, which makes up 15% of cases, and non-small cell lung cancer, which makes up 85% of all lung cancer cases (Sher et al., 2008). Non-small cell lung cancer comprises adenocarcinoma of the lung, squamous lung cancer, and large cell carcinoma, with adenocarcinoma of the lung being the predominant type, accounting for approximately 40% (Samson et al., 2007). Unfortunately, for the majority of patients with lung adenocarcinoma (LUAD), targeted therapy has not been successful, therefore, new early biomarkers and treatment options are urgently required.

Macrophages are specialized, long-lived, phagocytic immune cells that are derived from monocytes, which are in turn derived from bone marrow precursor cells. Along with neutrophils, they are the first line of defense in case of infection that take part in the identification, phagocytosis, and destruction of pathogens and cellular debris. Additionally, macrophages aid in antigen presentation to the T cells and the induction of costimulatory molecule production by other antigen-presenting cells, initiating adaptive immune responses (Rogler and Baumgart, 2017). In the early phases of inflammation, they contribute by releasing cytokines and chemokines, which in turn attract more immune cells to the area of inflammation. Based on their activation and function, macrophages can be broadly categorized into two subtypes: classically activated M1 macrophages and alternatively activated M2 macrophages. Several factors contribute to various phenotypes and macrophage activation states, including signaling molecules, epigenetics, transcription factors, growth factors, and post-transcriptional mechanisms and modifications, along with niche signals like cytokines, intercellular interactions, and metabolites (Chen and Zhang, 2017; T’Jonck et al., 2018; Collins et al., 2021). However, macrophage activation is important for inflammation, disease progression, and tissue homeostasis.

In addition to its regulatory function in combating diseases, macrophages also have a harmful role in chronic inflammation, autoimmune disorders, and cancer. In the conventional immune response, pro-inflammatory macrophages are suppressed, resulting in a reduction in their pro-inflammatory signaling. However, dysregulated macrophages continue to release inflammatory cytokines and signal in more immune cells during long-term damage which results in chronic inflammation and plays a fundamental part in the development and progression of tumors. After the development of the tumor, macrophages undergo a phase transition from an immune-active to an immune-suppressed state and are referred to as tumor-associated macrophages (TAMs). According to reports, the TAMs are present in high concentrations in lung adenocarcinoma and are associated with a poor patient prognosis (Zilionis et al., 2019; Qiao and Fu, 2022). Programmed cell death protein 1 (PD-1) has been reported to be expressed in a tumor-promoting isoform of tumor-associated macrophages that are formed during tumor progression from pre-invasive to invasive adenocarcinoma, controlling the lymphocyte-depleted microenvironment of invasive tumors, and protecting the tumor cells in the solid histological components of tumors (Garcia et al., 2022). Macrophages play a crucial role in the development of lung cancer, and their polarization status and severity of infiltration are closely related to patients’ prognosis. This study aimed to explore the construction of a clinical prediction model through macrophage-related genes that may help guide immunotherapy for lung adenocarcinoma patients and thus potentially improve their prognosis.

## Materials and methods

### Data acquisition and processing

The bioinformatics analysis in this study was supported by the Sangerbox platform (http://vip.sangerbox.com/) (Shen et al., 2022). The LUAD project’s clinical follow-up information and latest expression data were downloaded from The Cancer Genome Atlas (TCGA), which included sequencing data of expression profiles and SNV mutations identified by mutect2 processing (Colaprico et al., 2016). The final sample number of 500 TCGA datasets was achieved after scrutiny of the available data such as the exclusion of samples with missing clinical data, conversion of Ensembl to Gene symbols, and averaged when duplicate Gene symbols were encountered. The Gene Expression Omnibus (GEO) database was used to download four lung adenocarcinoma datasets with patient survival times. The accession numbers of the downloaded lung adenocarcinoma datasets were: GSE30219, GSE31210, GSE37745, and GSE50081. In addition to the same processing as the TCGA data, the removeBatchEffect function of the limma package was used (Ritchie et al., 2015) to remove batch effects between different GEO datasets (Supplementary Figure S1), and a final sample size of 300 GEO datasets was collected.

### Calculate macrophage scores and obtain positively associated genes

The Macrophage Score of LUAD samples and normal samples was calculated by TIMER. The Pearson correlation coefficients were calculated between Macrophage Score and protein-coding genes (PCGs) in tumor samples and then filtered at a threshold R > 0.4 and *p* < 0.05 (Li et al., 2017).

### Functional enrichment analysis

The functional enrichment analysis is used to enrich the biological functions involved in a large number of genes as a means of finding key pathways that influence the development of a disease. We used the clusterProfiler package and the org. Hs.eg.db package for the enrichment analysis (Yu et al., 2012). The species selected was *Homo sapiens* and the entries analyzed included all Gene Ontology (GO) and Kyoto Encyclopedia of Genes and Genomes (KEGG) entries, with *p*-values adjusted by False Discovery Rate (FDR).

### Construction of molecular isoforms

By employing univariate cox analysis using the coxph function in survival R package, the prognostic genes positively related to Macrophage Score were obtained. ConsensusClusterPlus was utilized to cluster the TCGA samples and cumulative distribution function (CDF) was used to determine the optimal number of clusters. A more stable clustering result was achieved by selecting the optimal number of clusters by assessing the Delta area curve of the CDF (Wilkerson and Hayes, 2010).

### Analysis of gene alterations

The SNVs of LUAD samples downloaded from TCGA were previously processed by mutect2 tool. The genomic characteristics including Number of Segments, Fraction Altered, Homologous Recombination Defects, and tumor mutation burden were obtained from previous research (Thorsson et al., 2018).

### Differences in clinicopathological, immunological, and chemotherapeutic characteristics between molecular subtype

The genomic alteration differences between molecular subtypes were further explored in the TCGA dataset. The mutation dataset was downloaded and screened for mutated genes with a mutation frequency of more than 3, using TCGA’s mutect2 software. With a selection threshold of *p* < 0.05, the Fisher’s exact test was performed in each subtype to screen for genes with significantly high-frequency mutations. In order to comprehend the changes in the immune microenvironment of patients’ subtypes, we assessed the level of immune cell infiltration in our TCGA cohort using the expression levels of immune cell gene markers. For the functional analysis of the scores of 28 immune cells (Charoentong et al., 2017), we employed single sample gene set enrichment analysis (ssGSEA) of gene set enrichment analysis (GSEA). Additionally, by using MCP-Counter, the scores of 10 immune cells were analyzed and the overall immune microenvironment infiltration score was estimated using ESTIMATE (Yoshihara et al., 2013). We analyzed the differences between subtypes of immunotherapy and the expression differences between immune checkpoints of subtypes were compared from the HisgAtlas database (Liu et al., 2017). Simultaneously, the TIDE (http://tide.dfci.harvard.edu/) software was used to assess the potential clinical effects of immunotherapy in pre-defined high and low subtypes (Jiang et al., 2018). For patients with Higher TIDE prediction scores, immunotherapy is less likely to be effective for them because of a higher probability of immune escape. We also analyzed the extent to which subtypes in the TCGA dataset responded to conventional chemotherapeutic agents, including Cisplatin, Erlotinib, Sorafenib, Dasatinib, Lapatinib, and AKT inhibitor VIII.

### Construction of prognostic models and validation

We further divided the GEO dataset into two parts according to the ratio Train: Test = 7:3 and performed the univariate COX regression analysis for Macrophage Score positive correlation gene to identify those with a greater prognostic impact (*p* < 0.001). Furthermore, lasso (least absolute shrinkage selection operator) regression was used in the TCGA dataset to further compress the risk model by reducing the number of genes. As the lambda grew, it was also noted that the number of independent variable coefficients increased and for model construction, 10-fold cross-validation was employed to investigate the confidence intervals at every respective lambda. Moreover, based on the screening genes in lasso analysis, stepwise multi-factor regression analysis by Akaike information criterion (stepAIC) was used, which considered the statistical fit degree of the model and the number of parameters used for fitting. In the MASS package, the stepAIC method was used which started from the most complex model and deleted one variable in turn to reduce AIC. A better model was obtained with a smaller value, which suggested that sufficient fitting degrees were obtained in the model with fewer parameters (Zhang, 2016).

Moreover, we calculated the RiskScore for each patient using the following formula: RiskScore = Σβi × Expi, where “Expi” refers to the level of gene expression of the prognosis-related gene signature and *β* is the Cox regression coefficient of the corresponding gene. Based on the median threshold, patients were distributed into two groups: high- and low-RiskScore groups. Employing the Kaplan-Meier technique, prognostic survival curves were produced and log-rank tests were performed to assess the significance of the differences.

Subsequently, a risk-related prognostic RiskScore was determined for each sample based on the formula defined by our sample risk score. Furthermore, using the R software package timeROC (Blanche et al., 2013), the receiver operating characteristic (ROC) analysis was performed of the prognostic RiskScore classification, where we studied the efficiency of prognostic classification of the training dataset for one, three, and 5 years respectively and the AUC line could be seen clearly in the area under of the model. The samples were divided into low- and high-risk groups by applying the median RiskScore as the cutoff and plotted KM curves. To confirm the robustness of the risk-associated genetic signature clinical prognostic model predictions, we performed validation on the TCGA lung adenocarcinoma validation dataset as well as the TCGA full dataset cohort and calculated the RiskScore for patients in the same way.

### RiskScore on different clinicopathological, immunological and chemotherapeutic characteristics

We grouped samples with distinctive clinical characteristics by comparing the distribution of RiskScore between clinicopathological characteristics subtypes and performed KM curve analysis. The same methods and data were used to explore the association between RiskScore and immunotherapy versus chemotherapy as in the molecular subtypes. Additionally, by selecting gene expression profiles that correspond to lung adenocarcinoma samples in the TCGA cohort and utilizing the R software package GSVA (Hänzelmann et al., 2013) to perform ssGSEA, the correlation between RiskScore and biological features was studied in various samples. The correlation between these features and RiskScore was then calculated and features that correlated at 0.5 or more were selected.

### RiskScore combined with clinicopathological features to further improve prognostic models

The most significant prognostic factors were analyzed based on univariate and multivariate Cox regression of RiskScore and clinicopathological characteristics in the TCGA cohort. For quantification of the risk assessment and probability of survival in patients with lung adenocarcinoma, we combined the RiskScore and other clinicopathological characteristics to create nomograms that identified the factors with the most significant impact on survival prediction from the model results. We further assessed the predictive accuracy of the model using calibration curves, and the reliability of the model was assessed using decision curve analysis (DCA).

## Results

### Characterization of lung adenocarcinoma macrophages and identification of related genes

**FIGURE 1:**
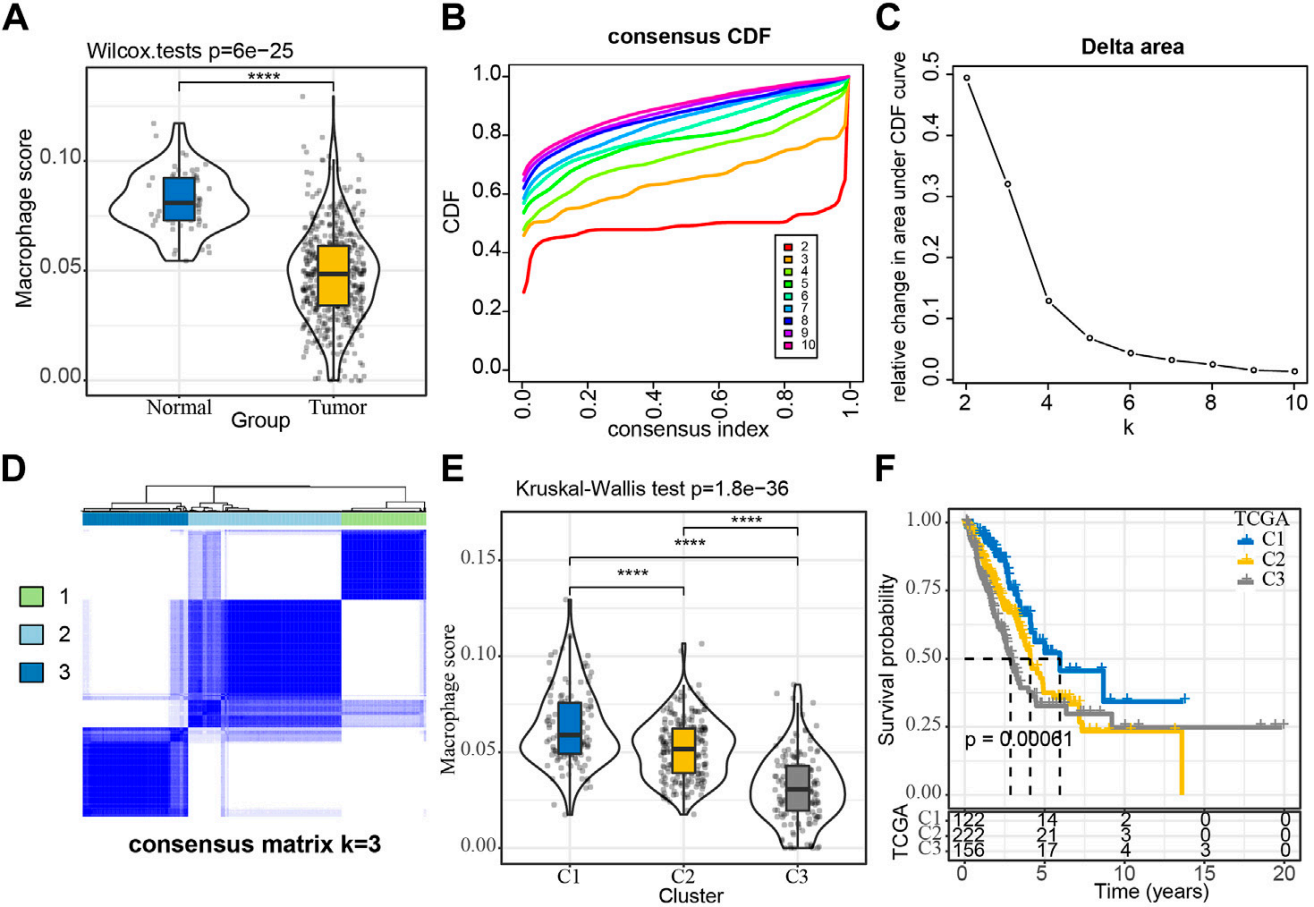
Construction of molecular subtypes using macrophage-associated prognostic genes **(A)** Comparison of Macrophage Score for normal and tumor samples; **(B, C)** TCGA cohort sample CDF curves and CDF Delta area curves and Delta area curve of consensus clustering, indicating the relative change in area under the cumulative distribution function (CDF) curve for each category number *k* compared with *k*- 1. The category number *k* is represented on the horizontal axis, and the relative change in the area under the CDF curve is shown on the vertical axis; **(D)** Sample clustering heat map at consensus *k* = 3; **(E)** Distribution of Macrophage Score among subtypes; **(F)** KM curves of the relationship between the prognosis of the three subtypes. ^****^ *p* < 0.0001.

We used TIMER software to assess the Macrophage Score in lung adenocarcinoma samples in the TCGA dataset and compared the difference between the tumor samples and the normal samples. The Macrophage Score was found to be substantially higher in the normal samples than in the tumor samples (Figure 1A). The Pearson’s correlation coefficients were also calculated between Macrophage Score and PCGs in the tumor samples and filtered for a threshold of R > 0.4 and *p* < 0.05 to obtain 1,044 genes positively correlated with the Macrophage Score. We performed GO/KEGG enrichment analysis on these 1,044 genes, and the GO function analysis annotated 679 BP (biological process) terms (FDR < 0.05), 80 MF (molecular function) terms (FDR < 0.05), and 100 CC (cellular component) terms (FDR < 0.05). GO enrichment was used to obtain some immune-related pathways. The KEGG pathway of different genes was enriched to 52 significant items (FDR < 0.05). Immune pathways such as Th1, Th2, and Th17 cell differentiation, and B cell receptor signaling pathways were also significantly enriched (Supplementary Figure S2).

### Molecular typing based on macrophage score positively related genes

We extracted 1,044 Macrophage Score for positively correlated genes from the TCGA expression profile matrix, and then performed univariate Cox analysis to obtain 65 genes associated with LUAD prognosis (*p* < 0.01), of which 1 gene was Risk gene (hazard ratio, HR > 1) and 64 were Protect genes (HR < 1) (Supplementary Figure S3A). The expressions of these 65 genes showed a significant positive correlation (Supplementary Figure S3B). We then clustered the TCGA samples by consistent clustering based on the 65 Macrophage Score for positively correlated genes and determined the optimal number of clusters based on the CDF. The analysis of the CDF Delta area curve revealed that stable clustering results were observed when the cluster is selected as 3. Finally, two molecular subtypes were obtained when we selected K = 3 (Figures 1B–D). The distribution of the macrophage scores of these three molecular subtypes was further analyzed and it was found that the C1 and C3 subtypes had the highest score and the lowest score respectively (Figure 1E). The prognostic characteristics of these three subtypes were also analyzed and significant differences in prognosis were observed, with C1 showing a better prognosis than the other two subtypes, whereas C3 had the worst prognosis (Figure 1F). In addition, we plotted the expression clustering heat map of 65 genes in different subtypes (Supplementary Figure S4). The C1 subtype exhibited significantly higher expression of these 65 genes and the C3 subtype displayed a low level of expression.

### Clinicopathological features between molecular subtypes

The differences in clinicopathological features were further explored between the different molecular subtypes in the TCGA-LUAD cohort. Different clinical features were compared across the three molecular subtypes and observed that the distribution of all features differed across the three subtypes, except for the M Stage. The largest proportion of the C3 subtypes was found in male patients aged >65 (Supplementary Figure S5).

### Mutational features between molecular subtypes

**FIGURE 2:**
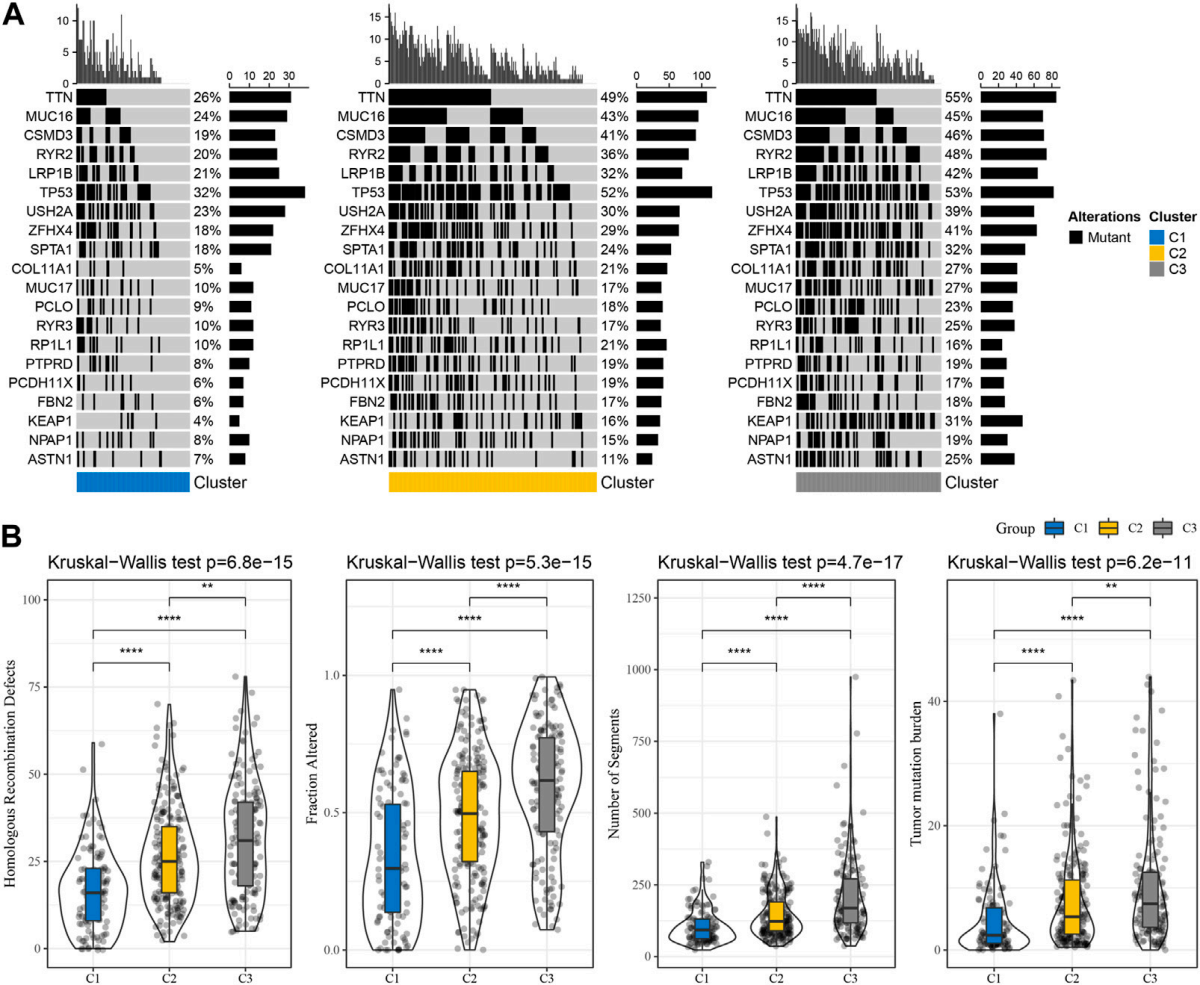
TCGA cohort data on the frequency of gene mutations and genomic changes in various subtypes. **(A)** Somatic mutation analysis (Fisher’s exact test) for different molecular subtypes; **(B)** Differences between different molecular subgroups in terms of homologous fraction altered, number of segments, recombination defects, and tumor mutation load. ^**^ *p* < 0.01, ^****^ *p* < 0.0001.

In the TCGA cohort, we further investigated the variations in genomic alterations between molecular subtypes. The mutation dataset was downloaded and processed by TCGA’s mutect2 software, and screened genes with a mutation frequency greater than 3. Within a total of 9,904 genes, 762 genes were identified to be significantly mutated in three subtypes (*p* < 0.05). The top 20 mutated genes were listed (Figure 2A). In addition, we compared the distribution of the Number of Segments, Fraction Altered, Homologous Recombination Defects, and tumor mutation burden between subtypes. These characteristics also differed among subtypes, suggesting a higher frequency of mutations in C3 and a worse prognosis for patients with more severe mutations (Figure 2B).

### Immunological characteristics between subtypes

**FIGURE 3:**
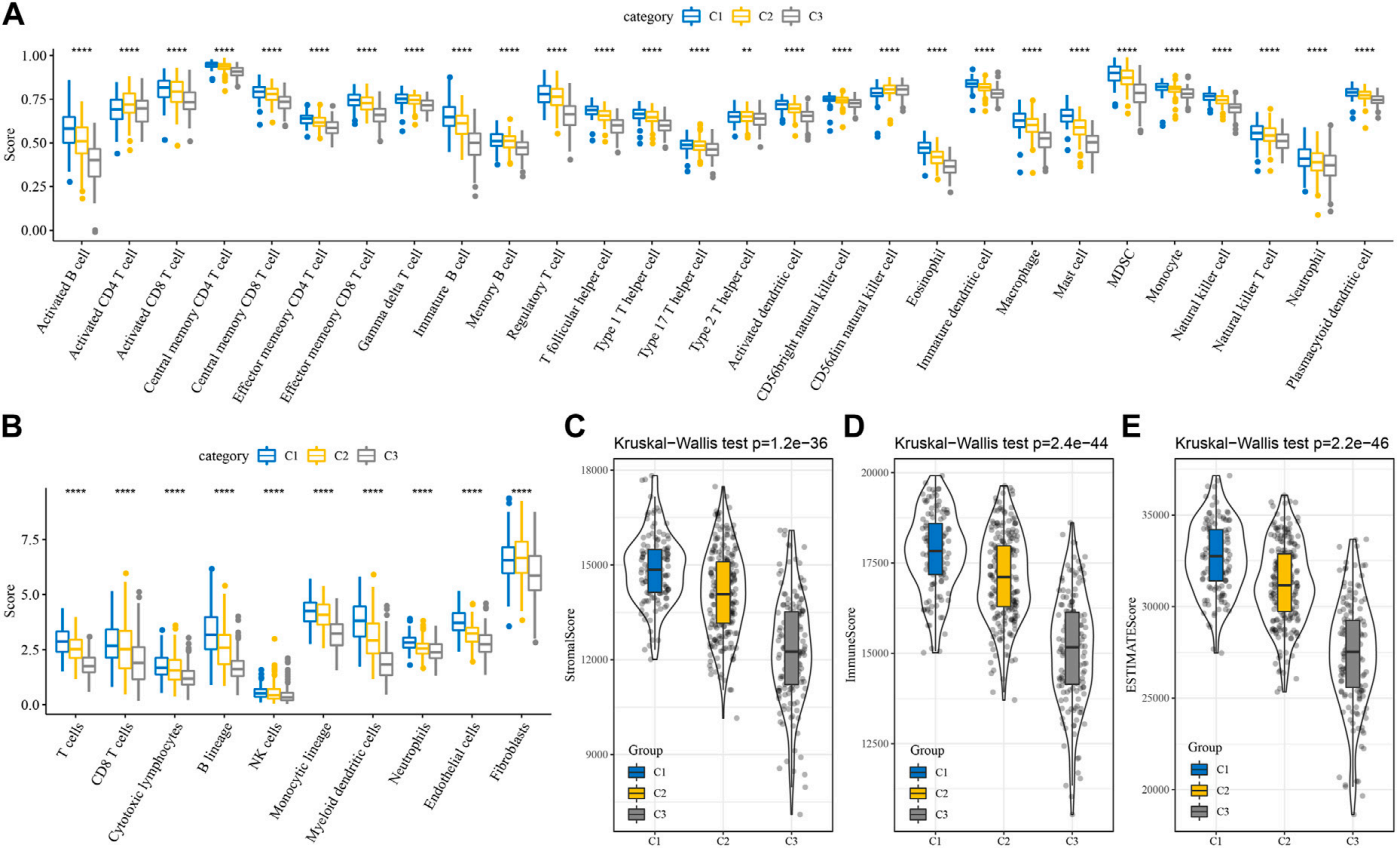
Differences of immune microenvironment in different subtypes. **(A)** Subtype comparison of 28 immune cell scores assessed by ssGSEA; **(B)** Subtype comparison of 10 immune cell scores assessed by MCP-Counter; **(C–E)** Subtype comparison of StromalScore, ImmuneScore, and ESTIMATEScore assessed by ESTIMATE. ^**^ *p* < 0.01, ^****^ *p* < 0.0001.

By measuring the degree of immune cell infiltration in the TCGA cohort using the expression levels of immune cell gene markers, the variations in the immune microenvironment of patients in the subtype were further elucidated. We further assessed the immune microenvironment infiltration scores using MCP-Counter, GSEA, and ESTIMATE. The results found the same trend of higher immune scores for C1 among the 28 immune cell types analyzed by GSEA (Figure 3A). In the MCP-Counter analysis, the immune score was higher for the C1 subtype among the 10 immune cell types (Figure 3B). Three scores for the ESTIMATE are consistent with MCP-counter and ssGSEA (Figures 3C–E). These results suggested that Immunosuppression was prevalent in patients with the worse prognosis in the C3 subtype, which was consistent with our previous macrophage immune score. It also suggested that the occurrence of immunosuppression may be an important factor in the development of immune escape in lung adenocarcinoma.

### Differences in immunotherapy/chemotherapy between subtype subtypes

**FIGURE 4:**
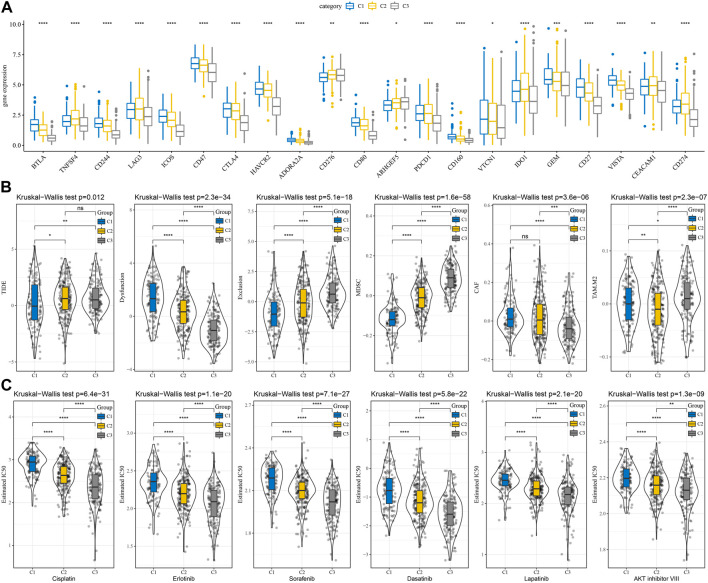
Differences in immunotherapy and chemotherapy scores between subtypes. **(A)** Immune checkpoints differentially expressed between subtypes in the TCGA cohort; **(B)** Differences in TIDE analysis results between subtypes in the TCGA cohort; **(C)** The box plots of the estimated IC50 for Cisplatin, Erlotinib, Sorafenib, Dasatinib, Lapatinib, and AKT inhibitor VIII in TCGA cohort. ^*^ *p* < 0.05, ^**^ *p* < 0.01, ^***^ *p* < 0.001, ^****^ *p* < 0.0001.

The differences in immunotherapy were analyzed and compared the differences in the expression of immune checkpoints between the subtypes. Most of the immune checkpoint genes were observed to be differentially expressed in each subtype (Figure 4A). The differences in immunotherapy between subtypes were studied by using the TIDE software, which evaluated the potential clinical effects of immunotherapy in the high and low subtypes. The highest TIDE scores in the TCGA-LUAD cohort were observed in the C2 and C3 groups, suggesting that these two subtypes are more likely to escape from immunotherapy. This is in line with our previous scenario of immunosuppression occurring in C2 and C3. Additionally, T cell exclusion scored highest in C3, suggesting that immune escape mechanisms were more active in the presence of low T cell infiltration. MDSC (myeloid-derived suppressor cell) and TAM. M2 were significantly enriched in the C3 subtype, indicating a high degree of tumor malignancy in C3 (Figure 4B). In addition, we also studied the subtypes’ response to conventional chemotherapeutic drugs in the TCGA dataset and observed that the C3 subtype was more sensitive to six drugs, Cisplatin, Erlotinib, Sorafenib, Dasatinib, Lapatinib, and AKT inhibitor VIII (Figure 4C).

### Construction of a prognostic model for lung adenocarcinoma

We further divided the datasets from GEO into two parts according to the ratio Train: Test = 7:3. No significant difference on survival information was observed between two groups (Supplementary Table S1). Then the univariate Cox regression analysis was performed for the genes associated with macrophage score, a total of 22 genes with a high prognostic impact were identified (*p* < 0.001), including 10 “Risk” and 12 “Protective” genes (Supplementary Figure S6A). Using lasso regression in the GEO dataset, the 22 genes were further compressed to reduce the number of genes in the risk model. The 10-fold cross-validation was used for model construction and the confidence intervals analysis at each lambda. The model was optimized at lambda = 0.0193, therefore, we selected 16 genes at lambda = 0.0342 as the next target genes (Supplementary Figures S6A–C). Based on the 16 genes from the lasso analysis, we further used stepwise multivariate regression analysis. Ultimately, eight genes were identified as relevant genes affecting prognosis, namely CTTNBP2NL, CYP2U1, FAM13C, CDH23, EXOC5, CD300A, MRO, ARHGEF6 (Supplementary Figure S6E).

### Development and validation of clinical prognostic models

**FIGURE 5:**
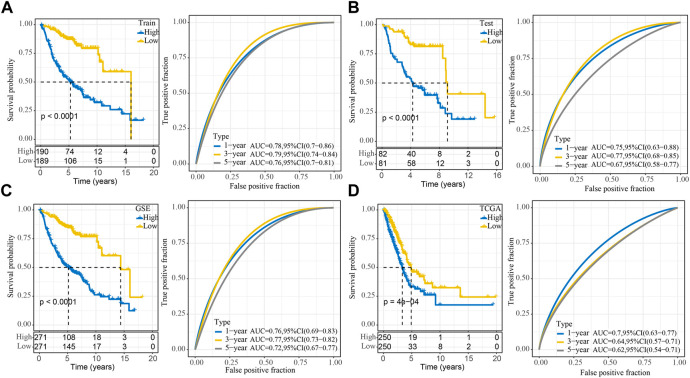
Stability of the RiskScore prognostic model for different datasets. **(A)** ROC and KM survival curves for RiskScore in the GEO training data cohort; **(B)** ROC and KM survival curves for RiskScore in the GEO validation data cohort; **(C)** ROC and KM survival curves for RiskScore in the GEO cohort and KM survival curves in the GEO cohort; **(D)** ROC and KM survival curves for RiskScore in the TCGA cohort.

For each sample, a risk-related prognostic risk score (RiskScore) was calculated on the basis of the formula defined by our sample risk score. The R software package timeROC was used to carry out the ROC analysis of the prognostic classification of the RiskScore. The prognostic classification efficiency of the training dataset was analyzed for one, three, and 5 years respectively, which revealed that the model had a high area under the AUC line. The samples were divided into two groups: high and low-risk groups using the median RiskScore as the cutoff and plotted KM curves, from which a significant difference was observed between the high- and low- RiskScore groups, with 190 samples classified as high-RiskScore and 189 samples as low-RiskScore groups. A low overall survival rate was observed in the patients with higher RiskScore in the training cohort (Figure 5A). The robustness of the risk-related gene signature clinical prognostic model was confirmed by performing validation on the GEO lung adenocarcinoma validation dataset as well as the GEO full dataset cohort, where we calculated the RiskScore scores of patients in the same way. We observed similar results as the training set in the validation cohort, with an unfavorable prognosis for high RiskScore and a better prognosis for low RiskScore (Figures 5B,C). We also performed validation on the independent dataset TCGA and observed similar results as the training set in the validation cohort, demonstrating the reliability of our results (Figure 5D).

### Performance of RiskScore scores on different clinicopathological features

The distribution comparison of RiskScores across clinicopathological characteristics revealed significant differences in the TCGA dataset for all clinical characteristics except M Stage, where RiskScores were not significantly different. In order to prove that our RiskScore grouping has a good survival effect in different clinical characteristics, we divided the samples with different clinical characteristics into groups and performed a KM curve analysis. The RiskScore was higher in males aged >65 and was positively correlated with TN and pathological stage, suggesting that the RiskScore has a meaningful effect on the extent of disease in patients with lung adenocarcinoma (Supplementary Figure S7). Furthermore, in the different subtype plots, the subtypes with higher RiskScore had a worse prognosis (Supplementary Figure S8).

### Relationship between RiskScore and immunity and pathway characteristics

**FIGURE 6:**
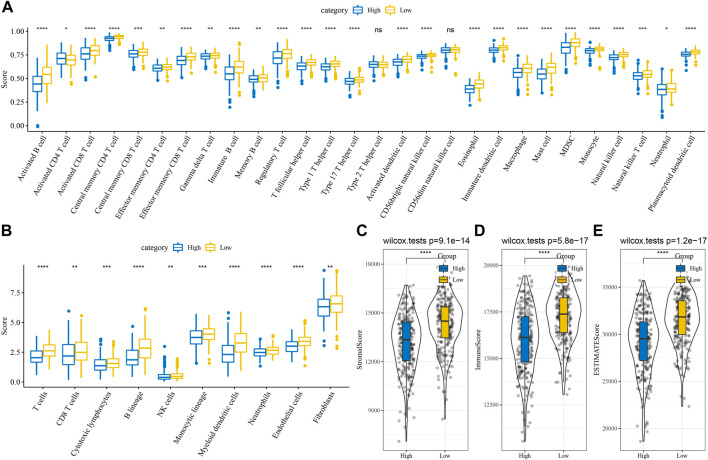
Differences of immune infiltration in different RiskScore groups **(A)** Subtype comparison of ssGSEA assessment of 28 immune cell scores; **(B)** Subtype comparison of MCP-Counter assessment of 10 immune cell scores; **(C)** Subtype comparison of ESTIMATE assessment of StromalScore; **(D)** Subtype comparison of ESTIMATE assessment of ImmuneScore; **(E)** Subtype comparison of ESTIMATE assessment of ESTIMATEScore. ns, not significant. ^*^ *p* < 0.05, ^**^ *p* < 0.01, ^***^ *p* < 0.001, ^****^ *p* < 0.0001.

The analysis of a difference in immunotherapy between the RiskScore groups was performed as well. 28 different immune cell types were studied by ssGSEA, and the results revealed that the low groups had a higher score than the high groups. (Figure 6A). The MCP-Counter was employed to examine 10 immune cell scores and observed higher scores in the low group (Figure 6B). The three scores assessed by ESTIMATE were consistent with ssGSEA and MCP-Counter, which suggested that immunosuppression also occurred in the high RiskScore group (Figures 6C–E). We calculated the correlation between RiskScore and 28 kinds of immune cell scores to note the correlation between RiskScore and immune function in different samples, and it was revealed that the RiskScore showed a negative correlation with 28 kinds of immune cell scores (Supplementary Figure S9A). In order to establish our understanding of the relationship between RiskScore and biological functions in various types of samples, the correlation between biological pathways and RiskScore was calculated. The functions with a correlation greater than 0.5 were chosen and found that RiskScore showed a significant positive correlation with KEGG_CELL_CYCLE, KEGG_DNA_REPLICATION, and other pathways (Supplementary Figure S9B).

### Differences in immunotherapy/chemotherapy between RiskScore subtypes

**FIGURE 7:**
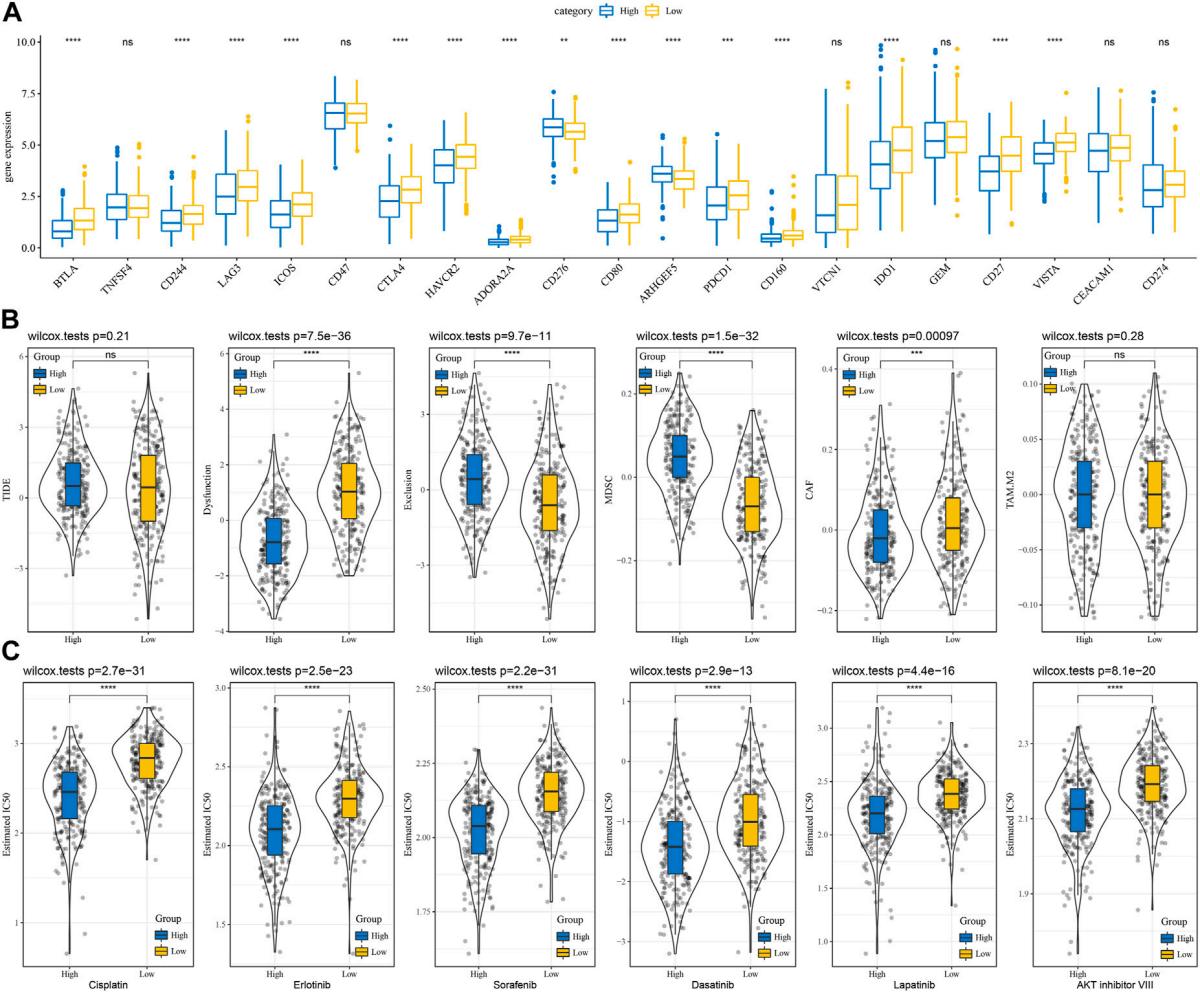
Differences in immunotherapy and chemotherapy scores between RiskScore groups. **(A)** Immune checkpoints differentially expressed between two risk groups in the TCGA cohort; **(B)** Differences in TIDE analysis results between two risk groups in the TCGA cohort; **(C)** The box plots of the estimated IC50 for cisplatin, Erlotinib, Sunitinib, Paclitaxel, Sorafenib, Crizotinib in TCGA cohort. ns, not significant. ^**^ *p* < 0.01, ^***^ *p* < 0.001, ^****^ *p* < 0.0001.

The differences between subtypes for immunotherapy were analyzed by comparing the expression of immune checkpoints which differed between subtypes (Figure 7A). Earlier, we found a negative correlation between RiskScore and the degree of T cell infiltration, while the Exclusion score demonstrates a higher Exclusion score in the group with a high RiskScore and at the same time a lower degree of T cell infiltration, indicating a more active occurrence of immune escape (Figure 7B). Moreover, the response of subtypes was analyzed in the TCGA dataset to conventional chemotherapeutic agents and observed that the high subtype was more sensitive to six drugs, Cisplatin, Erlotinib, Sorafenib, Dasatinib, Lapatinib, and AKT inhibitor VIII (Figure 7C).

### RiskScore combined with clinicopathological features to further improve prognostic models

**FIGURE 8:**
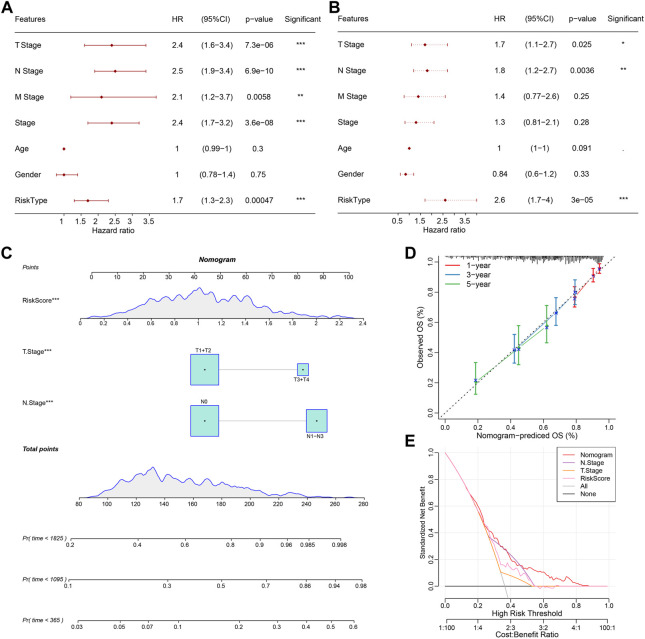
Validation of the predictive accuracy and reliability of the RiskScore prognostic model. **(A, B)** Univariate and multifactorial Cox analyses of RiskScore and clinicopathological features; **(C)** Nomogram model; **(D)** Calibration curves for 1, 3, and 5 years for the nomogram; **(E)** Decision curve (DCA) for the nomogram. ^*^ *p* < 0.05, ^**^ *p* < 0.01, ^***^ *p* < 0.001.

Univariate and multifactorial Cox regression analysis based on RiskScore and clinicopathological characteristics in the TCGA cohort showed RiskScore to be the most significant prognostic factor (Figures 8A,B). To quantify the risk assessment and probability of survival in patients with lung adenocarcinoma, we combined RiskScore and other clinicopathological characteristics to create a nomogram, and from the model results, we observed that RiskScore was the most impactful in survival prediction (Figure 8C). We further assessed the predictive accuracy of the model using calibration curves and observed that the predictive calibration curves at the 1, 3, and 5-year calibration points nearly overlapped with the standard curves, suggesting the good predictive performance of the nomogram (Figure 8D). Using DCA, the model’s reliability was also evaluated, and it was observed that the advantages of RiskScore and nomogram were considerably more than the extreme curves, with the nomogram showing the strongest predictive power for survival compared to other clinicopathological features (Figure 8E).

## Discussion

The relationship between cancer cells and the tumor microenvironment is complex and further studies are needed to predict the prognosis and improve clinical outcomes more accurately in patients with LUAD (Charoentong et al., 2017). We first explored the differences in the levels of macrophage infiltration between LUAD samples and normal samples in this study and found that macrophage infiltration scores were significantly reduced in diseased tissues. Concurrently, we explored a large number of genes positively correlated with macrophage infiltration scores and performed GO/KEGG enrichment analysis, finding that a large number of immune-related pathways were enriched, such as immune response, leukocyte differentiation, and B cell receptor signaling pathway. The expression of 1,044 genes positively associated with Macrophage Score was extracted and a univariate Cox analysis was performed for the identification of macrophage-related genes associated with lung adenocarcinoma prognosis. The 65 macrophage-associated genes with prognostic relevance to lung adenocarcinoma were clustered into three different subtypes of TCGA samples, with survival analysis revealing that subtype C1 had the best prognosis while C3 had the worst prognosis. In order to explore the factors affecting prognosis between the different subtypes, we explored the clinicopathological features, mutational features, and immunotherapy/chemotherapy features between different molecular subtypes. It was found that the subtype with a worse prognosis was predominantly male and older than 65 years and the frequency of mutations was significantly higher in the C3 subtype, which was likely to contribute to the poor prognosis of patients in the C3 subtype. The differences in the immune microenvironment of patients in the different subtypes were also discussed, and by using different algorithms we demonstrated that the subtypes with a worse prognosis were accompanied by more pronounced immunosuppression. The Exclusion score indicated the degree of activity of the immune escape mechanism in the presence of low T cell infiltration. Simultaneously, MDSC inhibited the body’s immune cells from performing normal innate and adaptive immune functions, and the score was significantly higher in the C3 subtype.

The GEO dataset was divided into two parts according to the ratio Train: Test = 7:3, and univariate Cox regression analysis and Lasso regression were performed to identify eight genes (CTTNBP2NL, CYP2U1, FAM13C, CDH23, EXOC5, CD300A, MRO, ARHGEF6) that had a strong prognostic impact. According to previous reports, CDH23, as a cell migration inhibitor, relaxed the adhesion ability of lung cancer cells through competitive binding and was negatively correlated with cancer metastasis (Sannigrahi et al., 2019). The RiskScore was calculated by the RiskScore scoring formula and patients were categorized into high- and low-RiskScore groups. Moreover, our RiskScore model indicated that a higher RiskScore was associated with a worse prognostic outcome. The RiskScore was also negatively correlated with the level of immune infiltration, with the individual immune infiltration algorithms showing that the group with a lower RiskScore exhibited significant immunosuppression, in line with our findings in the molecular subtypes.

Depending on their origin, lung macrophages are divided into tissue-resident macrophages (TRMs) and monocyte-derived macrophages (MDMs). TRMs exist before birth, independently of the adult hematopoietic system, and are locally self-renewing, coordinating tissue remodeling, and maintaining tissue integrity (Perdiguero and Geissmann, 2016). These two classes of TAMs have also been studied in tumor microenvironments, but their functions are different. MDMs inhibit tumor growth in the tumor microenvironment, while TRMs play an important role in tissue homeostasis and host defense. Studies have shown that M1-type macrophages are predominant in the early stages of lung cancer, while M2-type macrophages predominate in the mid to late-stage stages, with a gradual conversion from M1 to M2 phenotype as the tumor progresses (Qian and Pollard, 2010). Epithelial-mesenchymal transition (EMT) refers to the loss of polarity of epithelial cells, which take on the characteristics of mesenchymal cells. TAMs release cytokines i.e., IL-6, IL-10, and TGF- β, all of which regulate EMT. In addition, IL-6 and IL-10 can also induce M2 macrophage differentiation in an IL-4-dependent manner through activation of JAK/STAT3 (Dehai et al., 2014; Fu et al., 2017).

In summary, macrophages play a critical role in the development of lung adenocarcinoma, as well as in the prognostic outcome of patients. Therefore, it is important to further explore the impact of macrophage expression and related signaling pathways in lung adenocarcinoma. The RiskScore model in this study may provide new ideas for immunotherapy of lung adenocarcinoma and provide an important theoretical basis for tumor immune microenvironment therapy.

## Conclusion

Firstly, we identified significant macrophage suppression in lung adenocarcinoma, mined and enriched for macrophage-positive genes, constructed molecular subtypes based on prognostically relevant macrophage score positive genes, and analyzed the immunological and immunotherapeutic potential of the subtypes. In addition, we constructed the RiskScore clinical prognostic model, which is robust and independent of clinicopathological features and has stable predictive performance in independent datasets. Finally, we further improved the prognostic model by combining RiskScore with clinicopathological features, which had high predictive accuracy and survival prediction.

## Data Availability

The datasets presented in this study can be found in online repositories. The names of the repository/repositories and accession number(s) can be found in the article/Supplementary Material.
