# Supplementary figures and images for: Macrophage-related molecular subtypes in lung adenocarcinoma identify novel tumor microenvironment with prognostic and therapeutic implications
Source: Front Genet. 2022 Oct 3;13:1012164. doi: 10.3389/fgene.2022.1012164 (PMC9574025; doi:10.3389/fgene.2022.1012164)

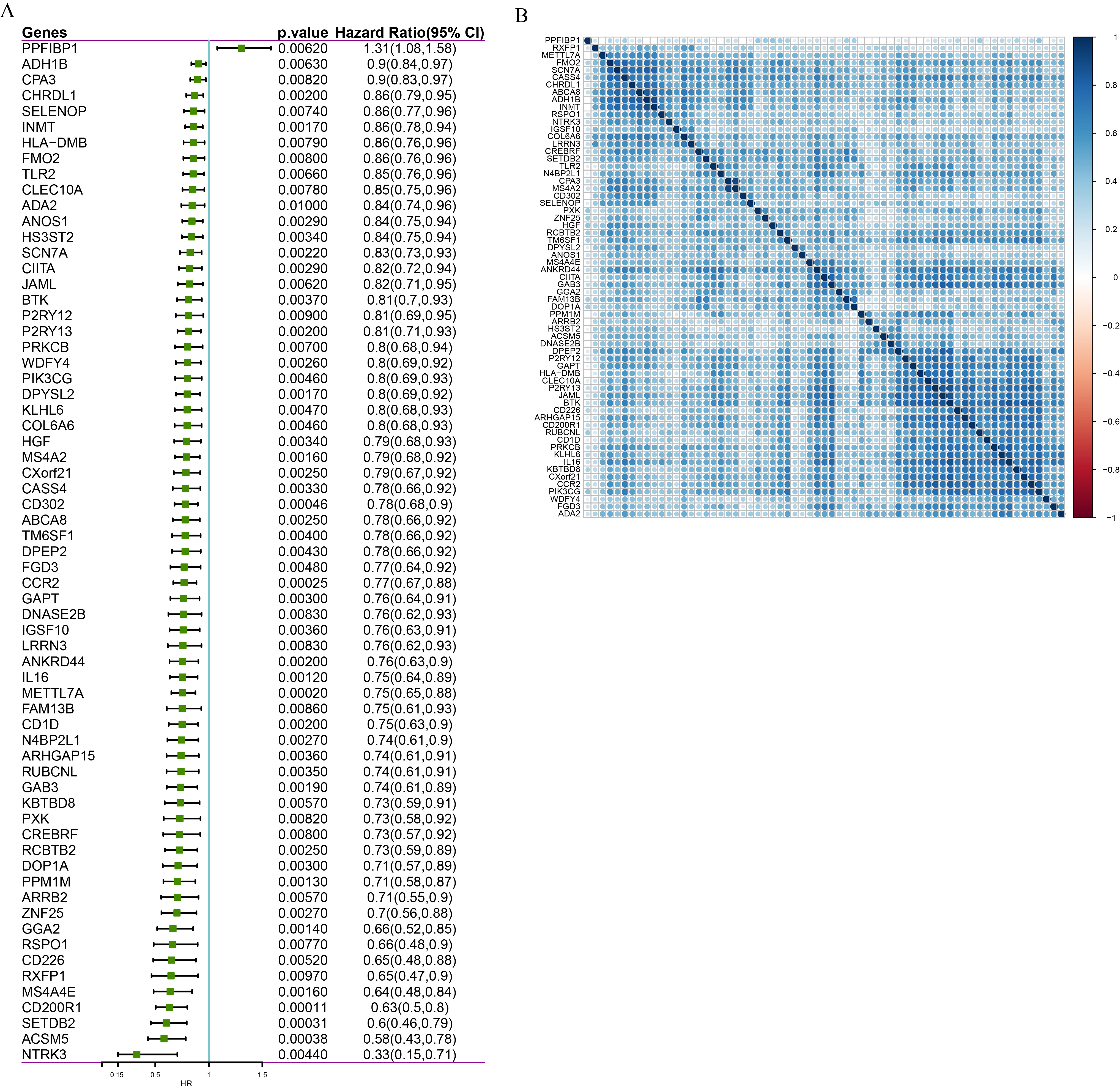

Supplement: Supplementary file 1 [file Image3.JPEG]

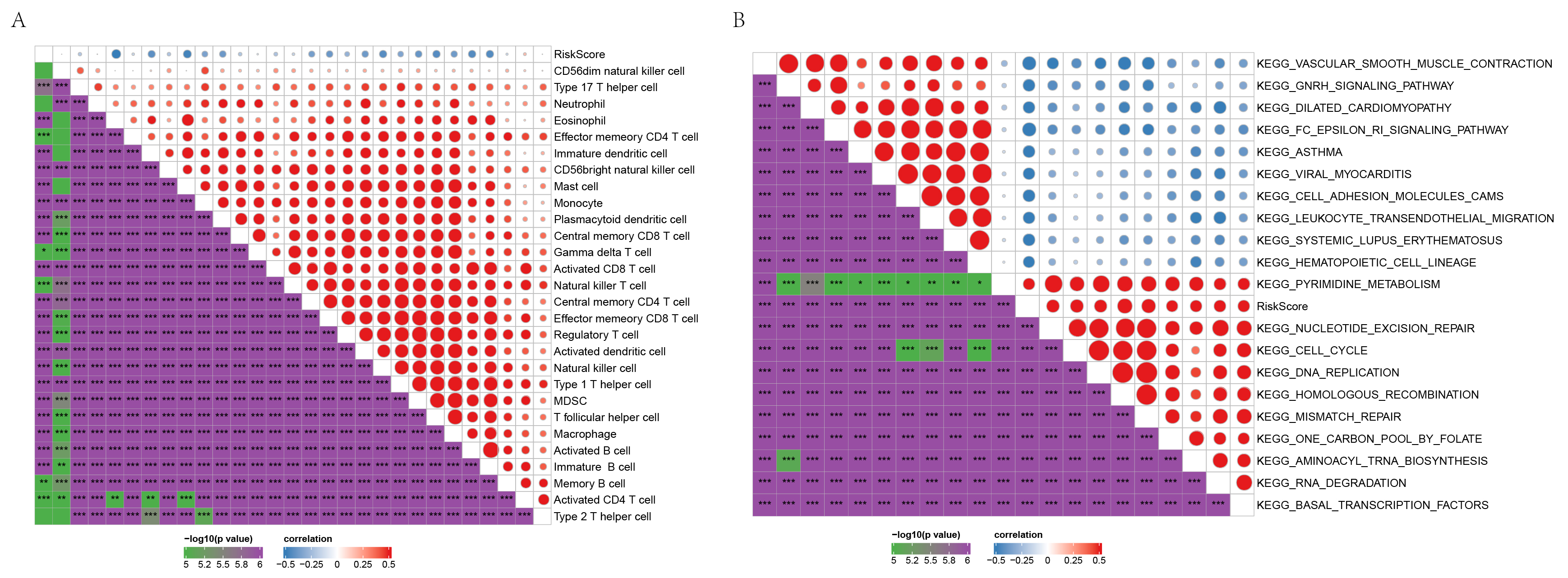

Supplement: Supplementary file 2 [file Image9.JPEG]

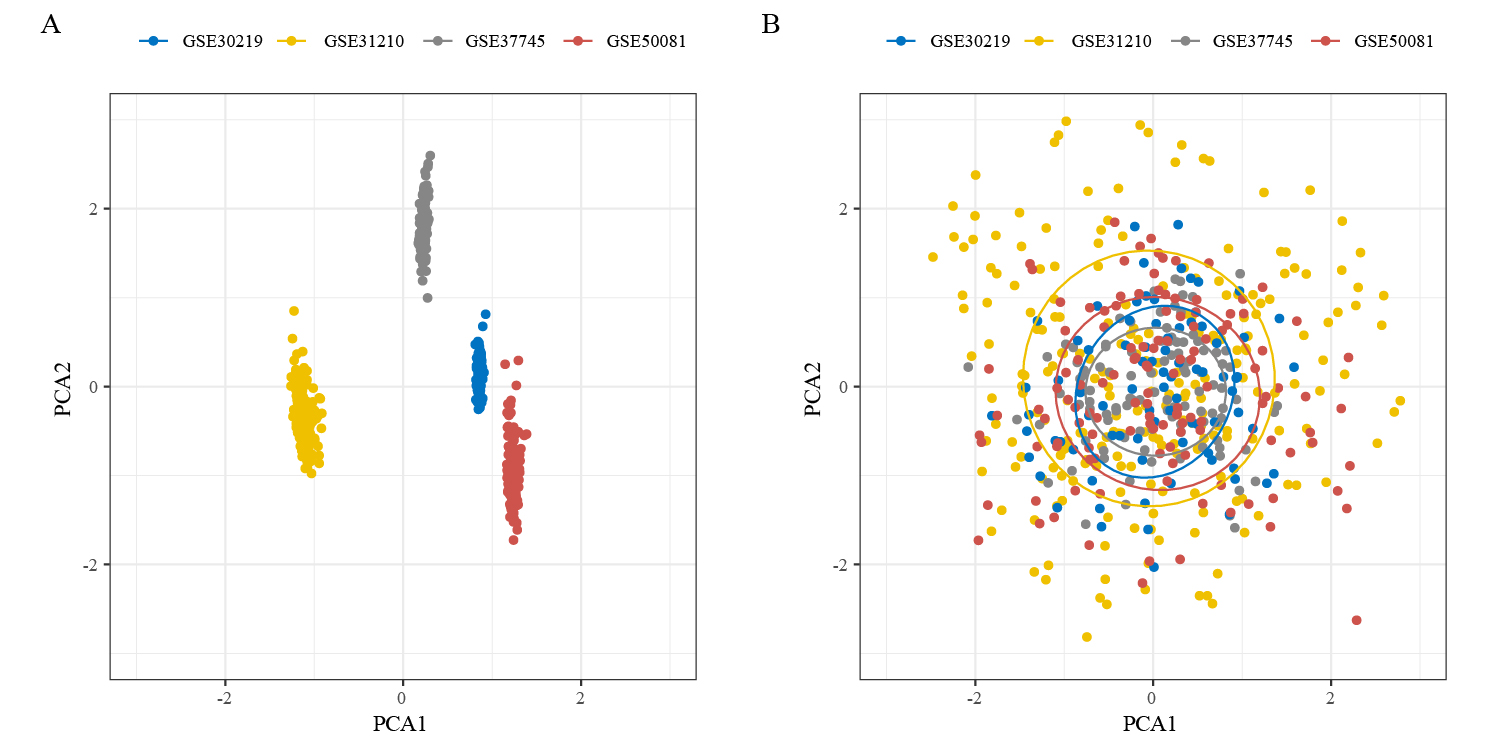

Supplement: Supplementary file 4 [file Image1.JPEG]

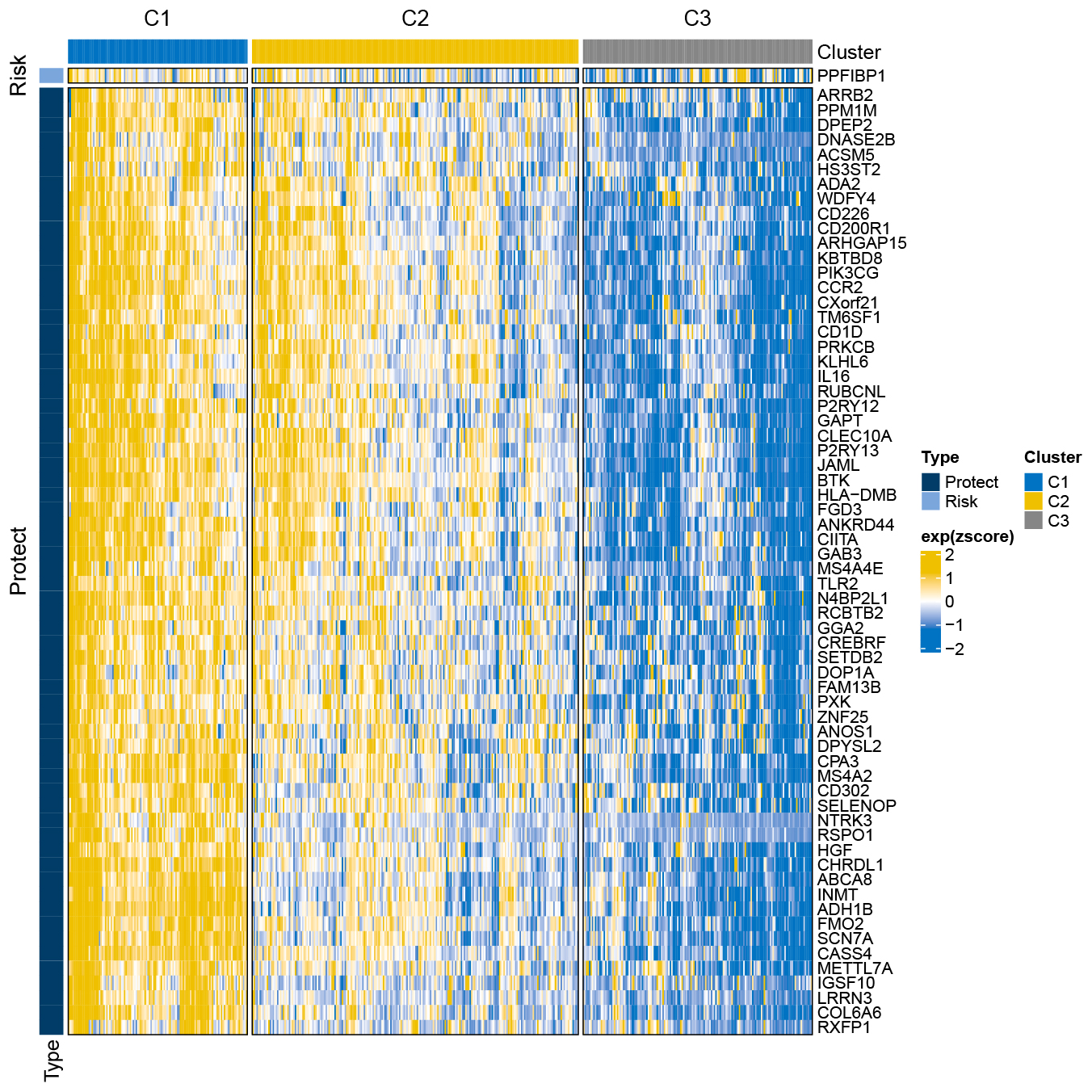

Supplement: Supplementary file 5 [file Image4.JPEG]

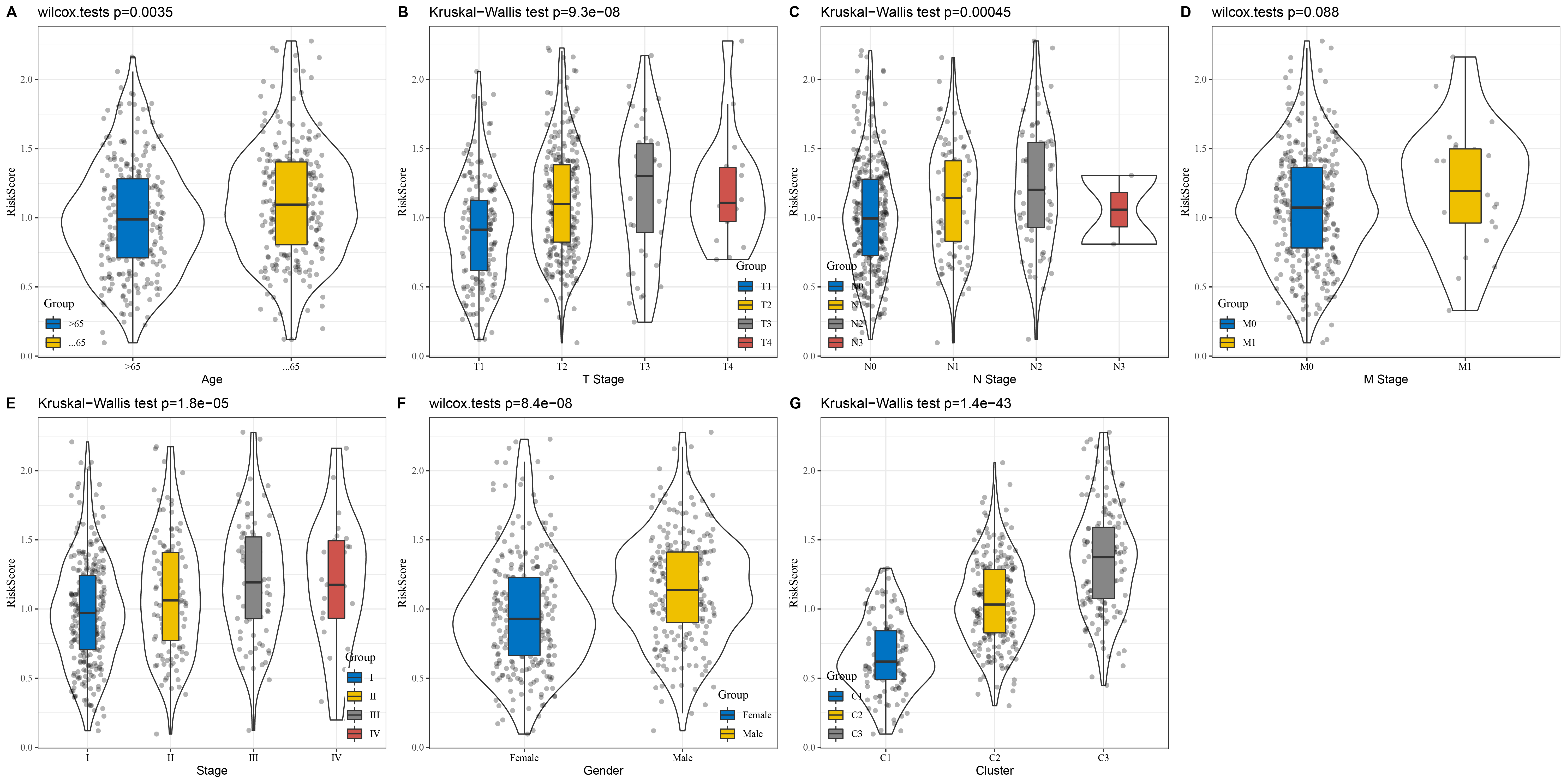

Supplement: Supplementary file 6 [file Image7.JPEG]

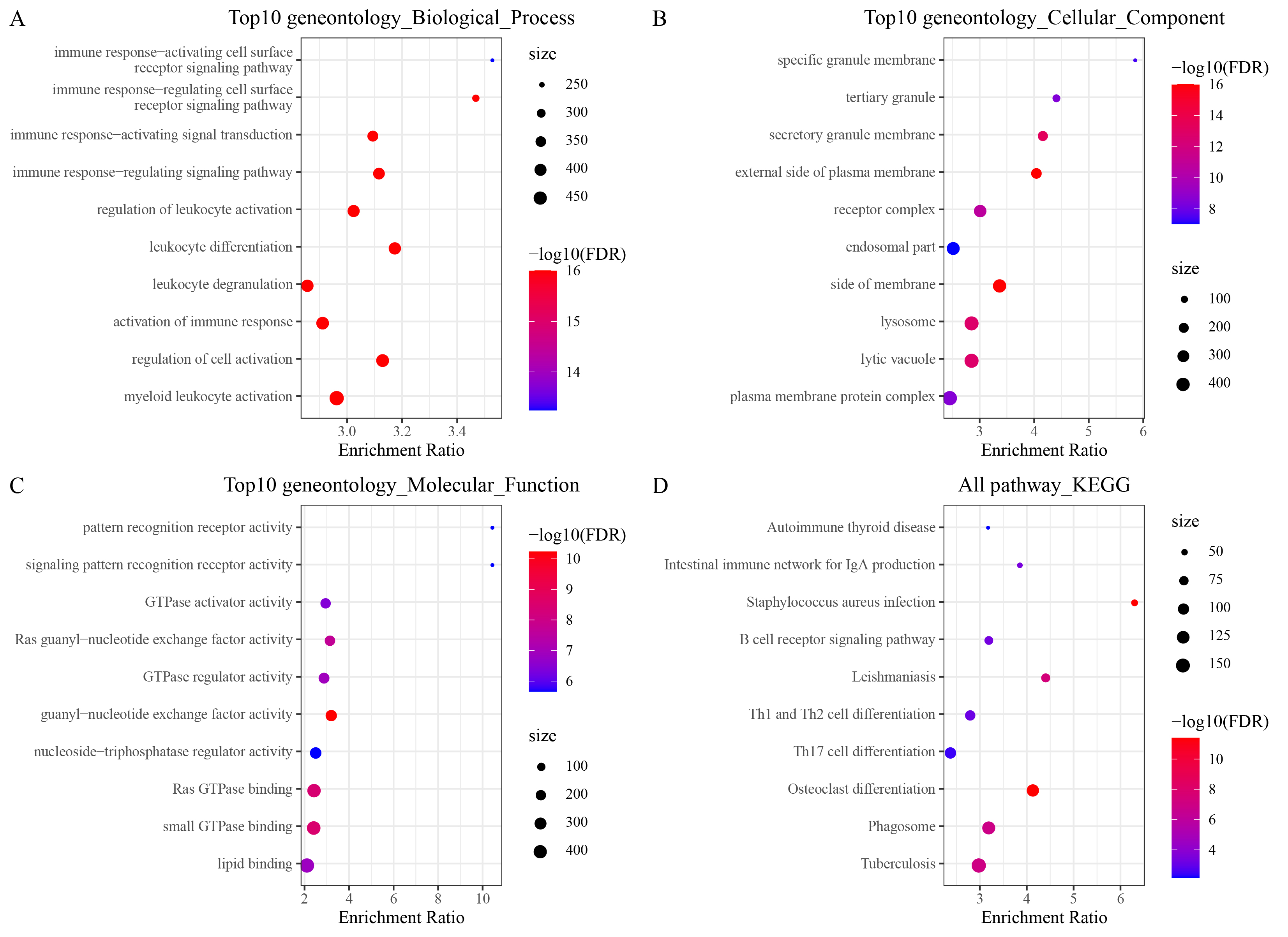

Supplement: Supplementary file 7 [file Image2.JPEG]

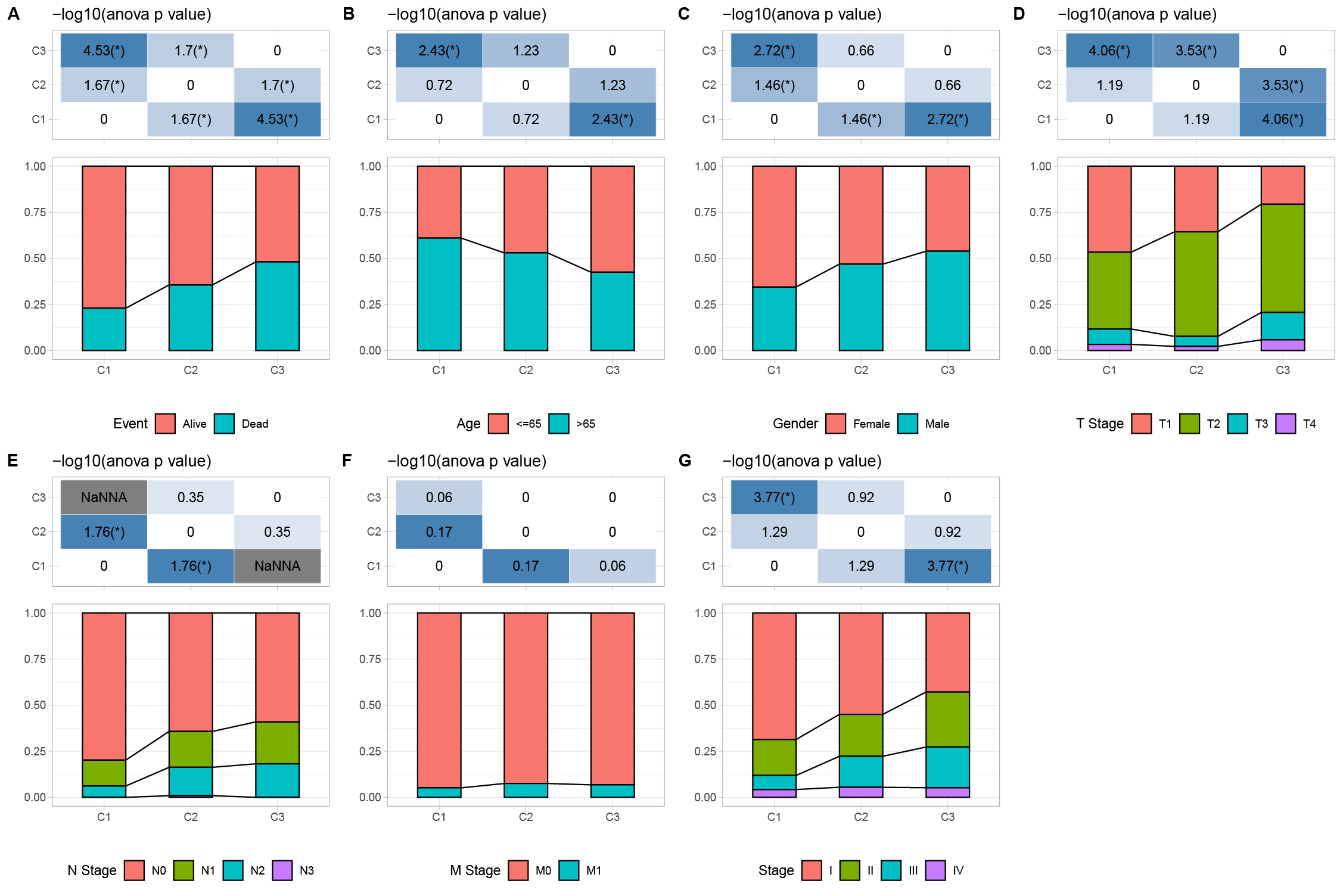

Supplement: Supplementary file 8 [file Image5.JPEG]

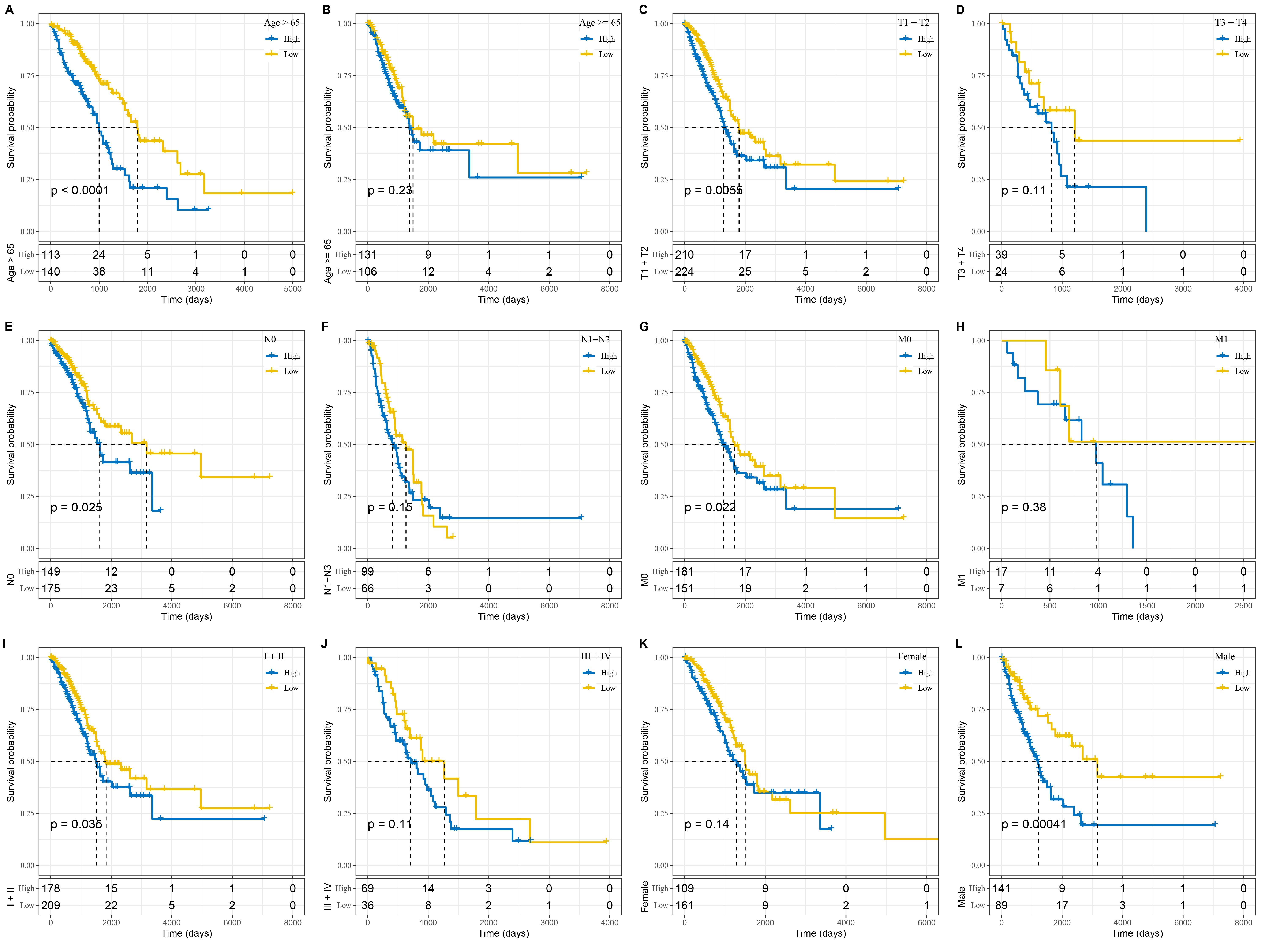

Supplement: Supplementary file 10 [file Image8.JPEG]

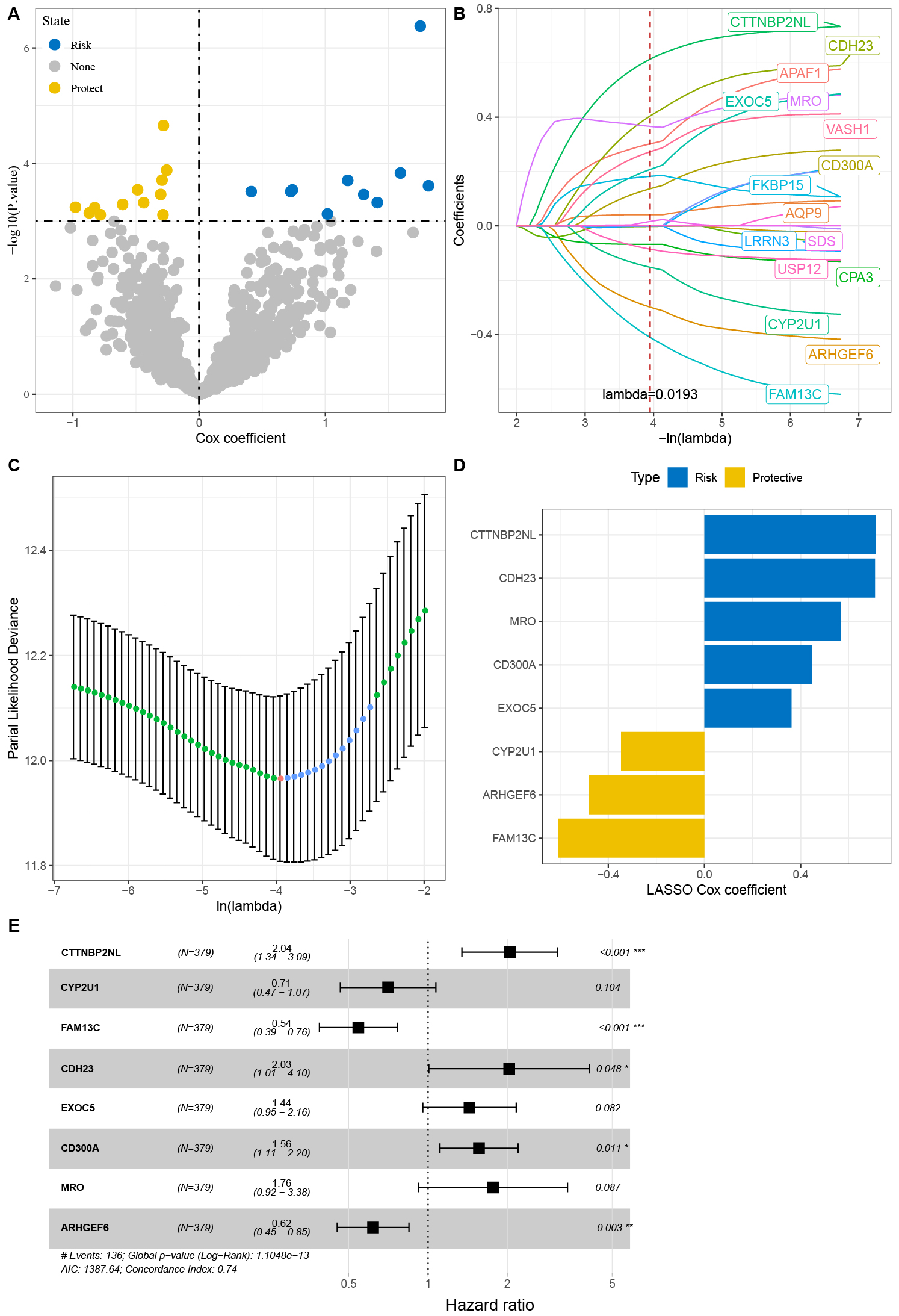

Supplement: Supplementary file 12 [file Image6.JPEG]
