# Supplementary material for: Macrophage-related molecular subtypes in lung adenocarcinoma identify novel tumor microenvironment with prognostic and therapeutic implications
Source: Front Genet. 2022 Oct 3;13:1012164. doi: 10.3389/fgene.2022.1012164 (PMC9574025; doi:10.3389/fgene.2022.1012164)
Supplement: Supplementary file 3 [file DataSheet3.docx]

For the data analyzed in this study please see:

https://www.jianguoyun.com/p/DSE5wVcQof7jChjDjM8EIAA
