# Supplementary material for: Macrophage-related molecular subtypes in lung adenocarcinoma identify novel tumor microenvironment with prognostic and therapeutic implications
Source: Front Genet. 2022 Oct 3;13:1012164. doi: 10.3389/fgene.2022.1012164 (PMC9574025; doi:10.3389/fgene.2022.1012164)
Supplement: Supplementary file 9 [file DataSheet2.docx]

Table S1. The comparison of clinical characteristics between the training and testing datasets.

|  | Test (N=163) | Train (N=379) | P-value |
| --- | --- | --- | --- |
| **OS.time** |  |  |  |
| Mean (SD) | 1730 (1070) | 1820 (1170) | 0.4205 |
| Median [Min, Max] | 1650 [61.0, 5710] | 1780 [6.00, 6630] |  |
| **OS** |  |  |  |
| Alive | 93 (57.1%) | 243 (64.1%) | 0.145 |
| Dead | 70 (42.9%) | 136 (35.9%) |  |
| **DataSet** |  |  |  |
| GSE30219 | 22 (13.5%) | 61 (16.1%) | 0.549 |
| GSE31210 | 71 (43.6%) | 155 (40.9%) |  |
| GSE37745 | 36 (22.1%) | 70 (18.5%) |  |
| GSE50081 | 34 (20.9%) | 93 (24.5%) |  |

Student t test was conducted for two groups on OS.time. Chi-square test was conducted for two groups on OS and DataSet.
