# Supplementary material for: Macrophage-related molecular subtypes in lung adenocarcinoma identify novel tumor microenvironment with prognostic and therapeutic implications
Source: Front Genet. 2022 Oct 3;13:1012164. doi: 10.3389/fgene.2022.1012164 (PMC9574025; doi:10.3389/fgene.2022.1012164)
Supplement: Supplementary file 11 [file DataSheet1.docx]

Supplementary figure legends

Figure S1. De-batching effects from the GEO dataset. A: Before de-batching. B: After de-batching.

Figure S2. GO/KEGG enrichment analysis. A: BP annotation map of Macrophage Score positively related genes. B: CC annotation map of Macrophage Score positively related genes. C: MF annotation of Macrophage Score positively associated genes. D: KEGG annotation of Macrophage Score positively associated genes.

Figure S3. A: Univariate analysis of Macrophage Score positively associated genes significantly associated with prognosis; B: Pearson correlation analysis between 65 prognosis-associated genes.

Figure S4. Heat map of the expression clusters of 65 macrophage-related prognostic genes in different isoforms.

Figure S5. Differences in clinical variable characteristics distribution throughout subtypes. A: Survival status characteristics of molecular subtypes of the TCGA cohort; B: Age characteristics of molecular subtypes of the TCGA cohort; C: Gender characteristics of molecular subtypes of the TCGA cohort; D: T Stage characteristics of molecular subtypes of the TCGA cohort; E: N Stage characteristics of molecular subtypes of the TCGA cohort. F: M Stage characteristics of molecular subtypes of the TCGA cohort; G: Stage characteristics of molecular subtypes of the TCGA cohort. (The lower half was the proportion and the upper half was the statistical significance of the difference between the two distributions-log10 (p-value).

Figure S6. Construction of the RiskScore prognostic model. A: A total of promising candidates were identified through the survival analysis of the Macrophage Score related; B: Trajectory of each independent variable with lambda; C: Confidence interval under lambda; D: Distribution of LASSO coefficients of the Macrophage Score related prognostic gene signature; E: Multifactor Cox forest plot of the eight genes in the GEO training set.

Figure S7. RiskScore differences within the TCGA cohort's different clinicopathological subtypes.

Figure S8. TCGA cohort survival KM curves for different clinicopathological subtypes.

Figure S9. Relationship between RiskScore and different immune cells and pathways. A: Correlation between 28 ssGSEA immune cell scores and RiskScore. B: Results of correlation analysis between TCGA data and KEGG pathways with a correlation greater than 0.5 with RiskScore.
